# Oral health status of children with autism spectrum disorder in KSA: A systematic review and meta-analysis

**DOI:** 10.1016/j.jtumed.2024.09.005

**Published:** 2024-09-20

**Authors:** Faris Y. Asiri, Marc Tennant, Estie Kruger

**Affiliations:** a Department of Preventive Dental Sciences, College of Dentistry, King Faisal University, Al-Ahsa, KSA; b International Research Collaboration—Oral Health and Equity, School of Allied Health, The University of Western Australia, Perth, WA, Australia

**Keywords:** اضطراب طيف التوحد, صحة الفم, أمراض الأسنان, نوعية الحياة المتعلقة بصحة الفم, المملكة العربية السعودية, Autism spectrum disorder, Dental diseases, KSA, Oral health, Oral-health-related quality of life

## Abstract

**Background:**

Individuals with autism spectrum disorder (ASD) often face challenges in maintaining good oral health, because of factors including sensory sensitivities, communication difficulties, and microbial imbalances in the oral cavity. Despite growing awareness of ASD, both in Kingdom of Saudi Arabia (KSA) and globally, no systematic review has comprehensively assessed the effects of ASD on oral health status in KSA.

**Objective:**

This study was aimed at assessing whether the oral health of individuals with ASD in KSA might differ from that of neurotypical individuals, on the basis of a systematic review framework.

**Materials and methods:**

According to the Participants, Exposure, Comparison, and Outcome (PICO) framework, a systematic search of electronic databases was conducted, and screening was independently performed by two reviewers. Conflicts were resolved through discussion. Data on study characteristics and oral health findings were independently extracted by the two reviewers. The risk of bias was assessed with the Joanna Briggs Institute (JBI) Critical Appraisal Checklist for Analytical Cross-Sectional Studies.

**Results:**

Of 763 initially identified articles, 14 met the inclusion criteria. These studies indicated that children with ASD have a higher prevalence of dental caries, greater gingival inflammation, and a greater risk of dental trauma than their neurotypical peers. Parents of children with ASD showed elevated concern regarding their children's oral health.

**Conclusion:**

Training dental professionals to manage patients with ASD is essential. Further research with larger sample sizes and rigorous methods is necessary to enhance understanding of the relationship between ASD and oral health outcomes in KSA.

## الملخص

**أهداف البحث:**

غالبا ما يواجه الأفراد المصابون باضطراب طيف التوحد تحديات في الحفاظ على صحة الفم الجيدة بسبب عوامل مثل الحساسيات الحسية، وصعوبات التواصل، والاختلالات الميكروبية في تجويف الفم. على الرغم من الوعي المتزايد باضطراب طيف التوحد في المملكة العربية السعودية والعالم، لم تقم أي مراجعة منهجية بتقييم تأثير اضطراب طيف التوحد على حالة صحة الفم في المملكة العربية السعودية بشكل شامل. تهدف هذه الدراسة إلى تقييم ما إذا كانت صحة الفم لدى الأفراد المصابين باضطراب طيف التوحد في المملكة العربية السعودية تختلف عن تلك الخاصة بالأفراد الطبيعيين عصبيا، وذلك باستخدام إطار المراجعة المنهجية.

**طريقة البحث:**

باستخدام إطار المشاركين والتعرض والمقارنة والنتيجة، تم إجراء بحث منهجي لقواعد البيانات الإلكترونية، وتم إجراء الفحص بشكل مستقل من قبل اثنين من المراجعين، مع حل النزاعات من خلال المناقشة. تم استخراج البيانات المتعلقة بخصائص الدراسة ونتائج صحة الفم بشكل مستقل من قبل اثنين من المراجعين. تم تقييم خطر التحيز باستخدام قائمة التقييم النقدي لمعهد جوانا بريجز للدراسات التحليلية المستعرضة.

**النتائج:**

من أصل 763 مقالة تم تحديدها في البداية، استوفى 14 منها معايير الاشتمال. أشارت هذه الدراسات إلى أن الأطفال الذين يعانون من اضطراب طيف التوحد لديهم معدل انتشار أعلى لتسوس الأسنان، وزيادة التهاب اللثة، وزيادة خطر الإصابة بصدمات الأسنان مقارنة بأقرانهم ذوي النمط العصبي. أبدى آباء الأطفال الذين يعانون من اضطراب طيف التوحد مخاوف متزايدة بشأن صحة فم أطفالهم.

**الاستنتاجات:**

تدريب أطباء الأسنان على إدارة المرضى الذين يعانون من اضطراب طيف التوحد أمر ضروري. هناك حاجة إلى مزيد من البحث باستخدام عينات أكبر ومنهجية صارمة لتعزيز فهم العلاقة بين اضطراب طيف التوحد ونتائج صحة الفم في المملكة العربية السعودية.

## Introduction

Autism spectrum disorder (ASD) is a complex neurodevelopmental condition characterized by persistent deficits in social communication and interaction, as well as restricted, repetitive patterns of behavior, interests, or activities.^1^ Although the exact cause of ASD remains elusive, it is widely accepted to result from a combination of genetic and environmental factors, including maternal age, gestational hypertension, and the use of certain medications during pregnancy.^2^ ASD is a spectrum disorder whose symptoms widely vary in severity and presentation. Clinically, ASD is often categorized into three levels, according to the level of support required.^3^ At level 1, individuals with ASD require some support but can generally function independently with assistance. They may experience mild difficulty in social interactions and communication, and although their behaviors might appear unusual, they typically do not severely disrupt daily life. At level 2, individuals require substantial support in their daily lives. They experience greater difficulty with social interactions, communication, and behavior. Their behaviors may be more repetitive and restrictive, and they may struggle to adapt to changes in routines. At level 3, individuals require substantial support and may experience major impairments in all areas of functioning. They experience severe difficulties with social interaction, communication, and behavior, and often require constant supervision and support.

ASD can present substantial challenges in maintaining good oral health.^4^ These challenges are attributable to various factors associated with the condition. First, the sensory sensitivities commonly experienced by individuals with ASD can make oral hygiene practices, such as tooth brushing and dental visits, extremely difficult.^5^ Sensory sensitivities might also lead to aversions to certain tastes, textures, or sensations, thus hindering tolerance to the sensation of toothbrushing or the taste of toothpaste.^5^ Second, the communication difficulties often associated with ASD can hinder understanding of oral hygiene instructions.^6^ Individuals with ASD may have difficulty in understanding the importance of oral health practices, and may struggle to follow instructions given by caregivers or dental professionals. Moreover, individuals with ASD may exhibit behaviors such as resistance to change, repetitive behaviors, or adherence to strict routines, thus further complicating oral hygiene practices. These behaviors may pose challenges in establishing and maintaining a regular oral hygiene routine. Additionally, individuals with ASD may be prone to certain microbial imbalances in the oral cavity, such as dysbiosis, thereby increasing the risk of dental issues such as dental caries and gum disease. Furthermore, some individuals with ASD may experience xerostomia (dry mouth), which can increase the risk of dental caries, because saliva plays a crucial role in maintaining oral health. Moreover, bruxism (tooth grinding) is more prevalent in individuals with ASD than the general population, and can lead to dental wear, jaw pain, and headaches.^7^

Globally, ASD affects approximately 1 in 160 children.^8^ In Kingdom of Saudi Arabia (KSA), the prevalence is similar to global rates, at approximately 2.5% among children 2–4 years of age, thus posing a substantial public health concern.^9^ Despite growing awareness of ASD both in KSA and globally, individuals with ASD often face challenges in maintaining good oral health, because of various factors including sensory sensitivities, communication difficulties, and microbial imbalances in the oral cavity.^9^ Although several published studies have described the effects of ASD on oral health status in the Saudi cohort, no systematic review had comprehensively assessed this relationship.^10^, ^11^, ^12^ Therefore, this systematic review was aimed at filling this gap by examining the existing literature on the effects of ASD on oral health status in KSA. By synthesizing available evidence, this review provides insights into the oral health needs of individuals with ASD in KSA, and may aid in identifying areas for future research and intervention, and informing current and future health policy in the country.

## Materials and Methods

### Focused question and protocol registration

By using the Participants, Exposure, Comparison and Outcome (PICO) framework provided in the Preferred Reporting Items for Systematic Reviews and Meta-Analyses (PRISMA) guideline,^13^ we created the following focused question: is the oral health (outcomes) of individuals (participants) with ASD (exposure) living in KSA better or worse than that in individuals without ASD or autism (controls)? The protocol was registered on PROSPERO before the start of the review (registration no. CRD42024517350). No methodological amendments were made to the protocol.

### Literature search

The search strategy was aimed at comprehensively gathering relevant literature from multiple databases without language restrictions. The databases searched included PubMed/MEDLINE, Embase, Scopus, and ISI Web of Science. In addition, Google Scholar was searched with a comprehensive search string designed to reflect the broader search strategy applied in traditional databases, to ensure that relevant studies indexed in Google Scholar but not in traditional databases were identified. The primary search was conducted during December 20–31, 2023, and the inclusion criteria were set to capture all studies published between database inception and January 1, 2024. A search update was conducted on March 15, 2024. Search terms were structured with free text words, MeSH terms, and Boolean operators (listed in Supplementary File S1). The following special care dentistry-related journals were manually searched: Community Dentistry and Oral Epidemiology; Special Care in Dentistry; and Journal of Disability and Oral Health. Additionally, the Saudi Dental Journal was manually searched to identify any additional relevant studies conducted within Saudi cohorts.

### Screening

Screening was conducted independently by two reviewers, and any conflicts were resolved through discussion with a third examiner. The consistency between examiners was assessed by calculation of inter-examiner reliability with the kappa statistic. The inclusion criteria for this review were studies reporting oral health status in children with ASD in KSA, including dental health indicators such as caries prevalence and relevant indices (e.g., DMFT/dmft scores), oral hygiene behaviors (e.g., brushing habits and dental flossing), gingival health indicators (e.g., gingivitis and periodontitis prevalence) and their associated indices (e.g., gingival index and plaque index), traumatic dental injury (TDI) prevalence, malocclusion (e.g., class I or class III), and oral-health-related quality of life (e.g., P-CPQ and OHRQOL). The review included original studies, such as randomized controlled trials, cohort studies, cross-sectional studies, and retrospective studies. The exclusion criteria included case reports, review studies, letters to the editor, commentaries, and studies not conducted in KSA.

### Data extraction

Before data extraction, a pilot form was developed to streamline the process. This form was used by two investigators for data extraction. Given that the initial inter-examiner reliability ratio exceeded 0.80, one investigator proceeded with the data extraction, whereas the other investigator reviewed and verified the data. A third examiner, a public health subject matter expert, was consulted for further validation as necessary. Information including study authors, year of publication, study design, city, setting (and number of centers), target population, characteristics of the population with ASD, variables/indices measured or recorded in individuals with ASD, and main oral health findings in individuals with ASD were extracted by two reviewers independently. Any disagreements were resolved by discussion.

### Risk of bias assessment

The Joanna Briggs Institute (JBI) Critical Appraisal Checklist for Analytical Cross-Sectional Studies^14^ was used to assess the risk of bias in the studies included in this review. This tool is particularly well aligned with our focus on assessing the prevalence and incidence of various oral health issues among children with ASD. The JBI tool's criteria are specifically tailored to address key aspects of cross-sectional studies, such as the appropriateness of the sampling frame, the validity and reliability of measurement instruments, and the adequacy of statistical analyses. These features make the tool a suitable choice for understanding disease burden and informing healthcare decisions in the context of this review.

### Meta-analysis and data synthesis

Among the included studies, we were able to pool the differences in mean scores of parental perception or concern regarding oral health between children with versus without ASD in two studies.^15^^,^^16^ The outcomes were pooled with a random effects meta-analysis, because of the high heterogeneity determined during a preliminary analysis in RevMan 5.4 software. Statistical significance was considered to be indicated by P < 0.05, and significant heterogeneity was considered to be indicated by I^2^ > 50%.^17^

## Results

### Literature search

**Figure 1:**
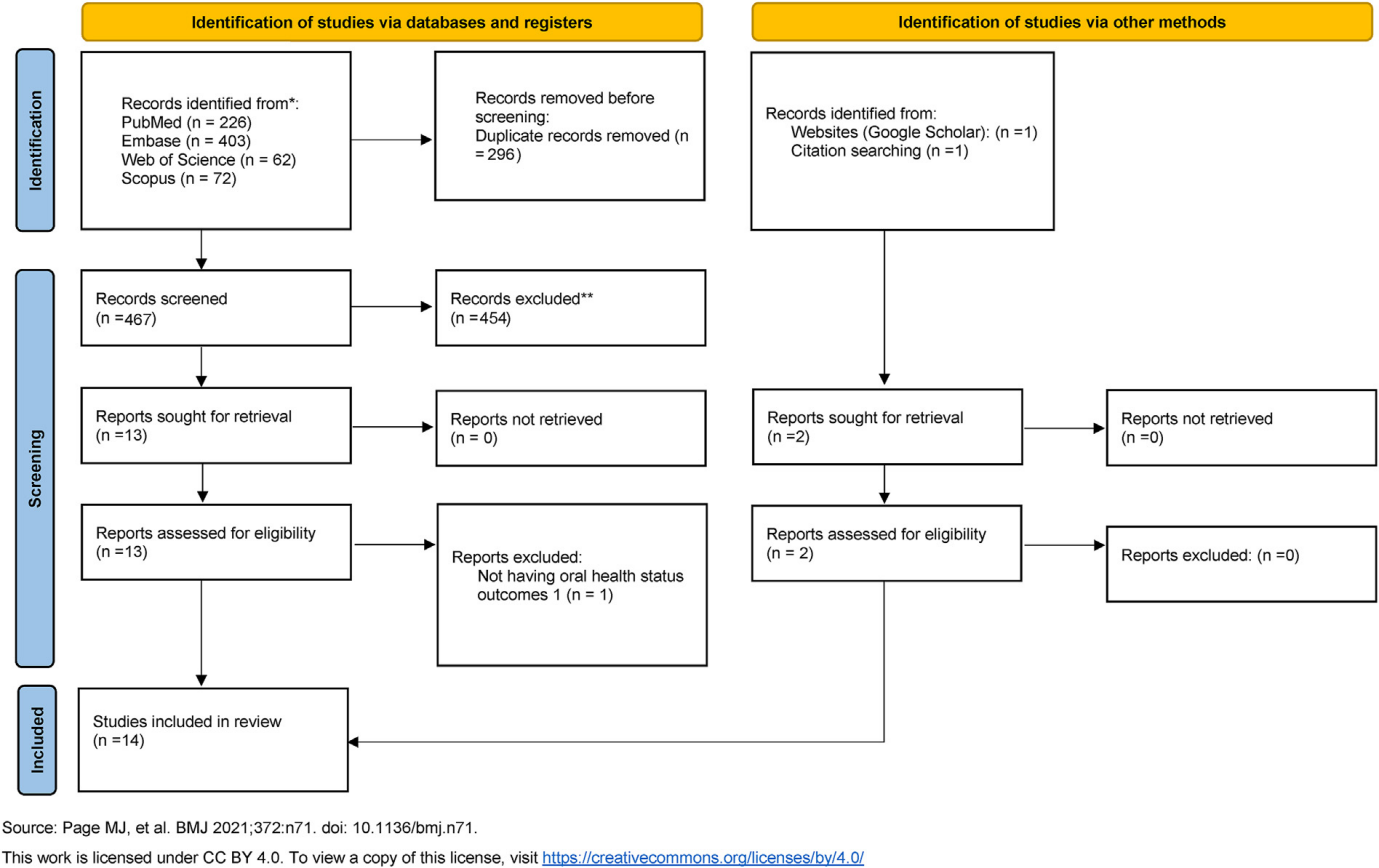
PRISMA flow diagram of the literature search used in this review.

The initial search of the main databases yielded 763 records. After removal of 296 duplicates, 467 articles were screened according to their titles and abstracts. Of these, 454 irrelevant studies were excluded, thus leaving 13 articles for full-text screening. One article was subsequently excluded because it did not report oral health status outcomes in individuals with ASD.^18^ Two additional articles were identified—one through cross-citation searching and another through an additional search in Google Scholar. Manual searching of special care dentistry-related journals did not yield any additional relevant studies for inclusion in this review. Therefore, 14 articles were included.^10^, ^11^, ^12^^,^^15^^,^^16^^,^^19^, ^20^, ^21^, ^22^, ^23^, ^24^, ^25^, ^26^, ^27^ The inter-examiner reliability score was calculated to be 0.86. The PRISMA flow diagram is illustrated in Figure 1.

### General characteristics of the studies

Thirteen studies were cross-sectional,^11^^,^^12^^,^^15^^,^^16^^,^^19^, ^20^, ^21^, ^22^, ^23^, ^24^, ^25^, ^26^, ^27^ and one was a retrospective cohort study.^10^ Six studies were conducted in Riyadh,^10^, ^11^, ^12^^,^^15^^,^^19^^,^^21^^,^^27^ two were conducted in Dammam,^22^^,^^23^ one was conducted in Makkah,^20^ two were conducted in Taif,^25^^,^^26^ and one was conducted in Jeddah.^16^ The settings varied across the studies: six studies were conducted in ASD-specialized schools,^10^^,^^20^, ^21^, ^22^, ^23^^,^^26^ five studies were conducted in rehabilitation centers,^11^^,^^12^^,^^16^^,^^19^^,^^27^ and three studies did not report the study setting.^,^^15^^,^^24^^,^^25^ In seven studies, parents of individuals with ASD were included,^11^^,^^12^^,^^15^^,^^16^^,^^22^^,^^24^^,^^27^ whereas in the other seven studies, only individuals with ASD were included.^10^^,^^19^, ^20^, ^21^^,^^23^^,^^25^^,^^26^ The number of individuals with ASD in each study ranged from 41 to 257, and their ages ranged between 3 and 18 years.^10^, ^11^, ^12^^,^^15^^,^^16^^,^^19^, ^20^, ^21^, ^22^, ^23^, ^24^, ^25^, ^26^, ^27^ In one study, the number of patients or any other demographic data were not included.^22^ The percentage of females with ASD, ranging between 24% and 52%, was reported in seven studies.^10^, ^11^, ^12^^,^^15^^,^^16^^,^^19^^,^^21^ The general characteristics of the included studies are reported in Table 1.

**Table 1:** General characteristics and outcomes reported in the included studies.

Study (author, year)	Design	City	Setting (n)	Target population	Characteristics of population with ASD (responders only)	Variables/indices measured or recorded in ASD	Main oral health findings in ASD
Pani 2013^15^	Cross-sectional	Riyadh	NR	Families of children with ASD	59 with ASD (vs 59 without ASD); 32.2% female; age: 8–13 years	P-CPQ	- Higher P-CPQ scores in children with ASD vs siblings; NSD in P-CPQ scores in children with vs without ASD. - ASD siblings had lower FL, EWB, and SWB scores compared to both ASD and non-ASD children, with NSD in OS scores.
Alaki 2016^16^	Cross-sectional	Jeddah	Rehabilitation centers (10); elementary schools (5)	Parents and children with or without ASD	75 with ASD (vs 100 without ASD); 31.6% female; age: 6–12 years	OHRQOL, dft/DMFT	- Daily life/parental concerns: children with ASD reported significantly more daily life problems and parental concerns than healthy children (p = 0.004, p = 0.008). - Oral well-being: children with ASD had significantly lower oral well-being scores than healthy children (p = 0.000–0.001). - Extra/intra-oral findings: children with ASD showed more extra-oral and intra-oral findings than healthy children (p = 0.000 for both). - Caries: children with ASD had greater prevalence (p = 0.013) and severity (p = 0.003) of caries than healthy children.
Alkhadra 2017^19^	Cross-sectional	Riyadh	Rehabilitation centers (5)	Patients with ASD	100; 35% female; age: 6–14 years	Malocclusion	- 40–41% class I. - 3–4% class III malocclusion.
Ashour 2018^20^	Cross-sectional	Makkah	Schools	Patients with ASD	41; 100% female; age: 6–11 years, 12–17 years	DMFT/dmft; diet and oral hygiene	Caries prevalence: 65.8%. Adjusted OR: 1.2.
Murshid 2014^11^^,^^12^^,^^a^	Cross-sectional	Riyadh	Multiple centers (3)	Patients with ASD and parents	344; 24.1% female; age: 3–14 years	OH habits (brushing); dental care visits	- Patients: 61.3% cannot brush teeth independently; 29.1% brush twice daily; 28.2% visit the dentist only in emergencies; 2% of parents believe the first dental visit should occur in the first year; 70.9% prefer high-sugar foods; 96.7% regularly consume soft drinks. - Brushing habits: 34.0% once daily, 29.0% twice daily, 28.8% irregularly. - Dental visits: 51.5% had no previous dental visits or treatment; 10.1% used nitrous oxide; ∼25% received treatment under general anesthesia; 48.5% used behavioral management techniques; 48.5% received treatment for dental problems.
Diab 2016^10^	Retrospective	Riyadh	School for special needs	Patients with or without ASD	50 with ASD (vs 50 without ASD); 52% female; age: 4–15 years	GI, PI	- Gingival inflammation/oral hygiene: children with ASD showed significantly higher gingival inflammation and poorer oral hygiene than children without ASD (p < 0.005 for both).
Al-Sehaibany^21^	Cross-sectional	Riyadh	Special needs schools (n = 3) vs mainstream schools (n = 3)	Patients with or without ASD	514 with ASD (vs 257 without ASD); 30% female; age: 4–15 years	TDI	TDI prevalence: higher prevalence of TDIs was observed in children with ASD (25.7%) than without ASD (16.3%) (p < 0.05).
Kotha 2018^22^	Cross-sectional survey	Dammam	Special needs schools (n = 3)	Parents of children with ASD	NR; NR female; NR	dmft, OH behavior, dental care utilization	Dental caries and habits: greater sugar intake between meals was associated with more caries; mean dmfs: 3.42 (males), 4.55 (females); 85.2% required brushing assistance; 73.8% were assisted by mothers; 49.2% had never visited a dentist; 36.1% visited only when having a problem; no significant effect of oral hygiene or parents' education on caries was observed.
AlHumaid 2020^23^	Cross-sectional	Dammam	School for special needs (n = 13)	Patients with ASD	75; NR female; age: 6–18 years; mean: 10.8 years	DMF, GI, PI, OH behavior	- Dental caries: prevalence: 76% in primary teeth, 68% in permanent teeth; mean dmfs: 0.85 ± 1.9 (primary), 1.03 ± 2.9 (permanent). - Gingival disease: 31 participants had gingival disease; mean gingival index: 1.03 ± 0.88; mean plaque index: 0.95 ± 0.43. OH behaviors: 22.7% did not brush; 61.3% did not floss; 24% always consumed sugar; positive parental attitude led to lower sugar intake and better oral health.
Al Hammad et al., 2020^24^	Cross-sectional	NR	NR	Parents of children with ASD	263; NR female; NR	Oral hygiene habits	Never brush teeth: 29.7%; brush twice per day: 22.1%; daily sugar/soft drink/dessert intake: 29.3%; dental clinic visit upon child's complaint: 41.4%.
Mohamed et al., 2021^25^	Cross-sectional	Taif	Not stated	Children with ASD and other disabilities	107 with ASD (with or without obesity); NR female; age: 6–16 years	OH status (dmft or DMFT)	High caries prevalence in children with ASD (78.5%) comparable to other disabilities.
Basha et al., 2021^26^	Cross-sectional	Taif	Schools (n = 25)	Children with ASD and other disabilities	74; NR female; age: 6–16 years	TDIs	Lower TDI prevalence (14.9%) in ASD than other conditions.
Alqahtani et al., 2023^27^	Cross-sectional study	Riyadh	NR	Parents of children with ASD	206; NR female; age: 3–12 years	Oral hygiene habits	50.5% brush teeth twice daily; 55.8% attend dental checkups.

ASD = autism spectrum disorder; P-CPQ = Parental Perception of Child Oral Health-Related Quality of Life Questionnaire.

FIS: Family Impact Scale; OS = oral symptoms; fl = functional limitations; ewb = emotional wellbeing; swb = social wellbeing; nsd = nonsignificant difference; SD = standard deviation; DMFT = decayed, missing, and filled teeth; GI = gingival index; PI: plaque index; OHRQOL; oral-health related quality of life; TDI = traumatic dental injuries; NR = not reported.

a Two studies on a single cohort have been grouped for clarity.

### Outcome measures

In five studies, the self/home oral hygiene habits of the included participants were analyzed.^11^^,^^12^^,^^22^^,^^25^ Dental caries indices were assessed in five studies.^16^^,^^20^^,^^22^^,^^23^^,^^25^ In one study, malocclusion was assessed,^19^ whereas in another study, TDIs were compared between children with versus without ASD.^21^ and other disabilities.^26^ Additionally, periodontal indices (plaque index and gingival index) were assessed in two studies.^10^^,^^23^

### Summary of results

#### Parental perception of oral-health-related quality of life

**Figure 2:**
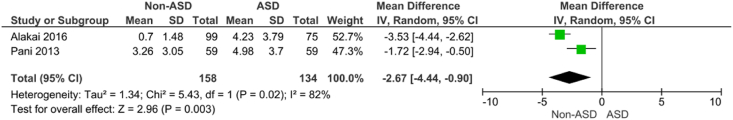
Forest plot of the parental perception or concern scores reported in two studies.

As shown in Figure 2, the results across two studies indicated that parents of children with rather than without ASD have greater concern regarding overall oral health (P = 0.003). However, significant heterogeneity was detected across the studies (I^2^ = 82%).^17^

#### Prevalence of dental caries

Ashour (2018) found a caries prevalence of 65.8% among children with ASD and identified a strong relationship between ASD status and caries.^21^ Similarly, Alaki (2016) observed a significantly higher prevalence of caries among children in the autism group (80%) than the control group (62.6%), with a p-value of 0.013.^16^ Kotha (2018) also observed that higher frequency of sugar intake between meals was associated with greater occurrence of dental caries.^22^ Furthermore, AlHumaid reported a high prevalence of dental caries in both primary (76%) and permanent teeth (68%) among children, thereby emphasizing the importance of positive parental attitudes in decreasing sugar intake and improving oral health^23^ Al Mohamed et al. reported that children with ASD had a 78.5% prevalence of dental caries, which was comparable to other disabilities.^25^

#### Oral hygiene care

Murshid (2014) highlighted inadequate oral hygiene care among children, including a substantial portion of children unable to brush their teeth independently.^11^^,^^12^ Only a minority brushed twice daily, and a substantial percentage received assistance during brushing, primarily from their mothers. The findings from AlHumaid et al. provided further support, by indicating that a considerable proportion of children did not brush regularly, and a substantial number did not use dental floss.^23^ Additionally, Alqahtani et al. reported that nearly half the children did not brush their teeth twice daily, and 22.30% indicated using dental floss.^27^

#### Periodontal indices (PI and GI)

Diab et al. reported significantly higher gingival inflammation and poorer oral hygiene among children with rather than without ASD.^10^ Similarly, AlHumaid et al. found that a considerable number of participants had gingival disease and a notable mean gingival index.^23^

#### Traumatic dental injuries

Children with rather than without ASD are at greater risk of TDIs. Al-Sehaibany reported a significantly higher prevalence of TDIs among children with rather than without ASD.^21^ However, in another study, the risk of TDIs was lower in children with ASD (14.9%) than in children with other disabilities.^26^

#### Risk of bias assessment

The detailed results of the risk of bias assessment are presented in Table 2. Overall only two studies were estimated to have a low risk of bias.^15^^,^^20^ Six studies were estimated to have a risk of bias,^10^^,^^19^^,^^22^^,^^23^^,^^25^^,^^27^ and six were graded as having a moderate risk of bias.^11^^,^^12^^,^^16^^,^^21^^,^^24^^,^^26^

**Table 2:** Results of quality assessment of the included studies.

Study (author, year)	Inclusion criteria	Description of participants and settings	Measurement of condition	Criteria for measurement	Confounding factors	Adjustment for confounding	Reliability of outcome measurement	Statistics	Risk of bias
Pani 2013^15^	Yes	Yes	Yes	Yes	Yes	Yes	Yes	Yes	Low
Alaki 2016^16^	Yes	Yes	Yes	Yes	No	No	Yes	Yes	Moderate
Alkhadra 2017^19^	No	No	Yes	Yes	No	No	No	No	High
Ashour 2018^20^	Yes	Yes	Yes	Yes	Yes	Yes	Yes	Yes	Low
Murshid 2014^11^^,^^12^^,^^a^	Yes	Yes	Yes	No	No	No	Yes	No	Moderate
Diab 2016^10^	No	Yes	Yes	Yes	No	No	No	No	High
Al- Sehaibany^21^	No	Yes	Yes	Yes	No	No	Yes	No	Moderate
Kotha 2018^22^	No	Yes	Yes	No	No	No	Yes	No	High
AlHumaid 2020^23^	No	No	Yes	No	No	No	No	No	High
Al Hammad et al., 2020^24^	Yes	Yes	Yes	No	No	No	Yes	No	Moderate
Mohamed et al., 2021^25^	No	Yes	Yes	Yes	No	No	No	No	High
Basha et al., 2021^26^	No	Yes	Yes	Yes	No	No	Yes	No	Moderate
Alqahtani et al., 2023^27^	No	Yes	Yes	Yes	No	No	No	No	High

a Two studies on a single cohort have been grouped for clarity.

## Discussion

Despite the limitations of this review, we observed that the existing research indicates that Saudi children with ASD have a higher prevalence of dental caries than their neurotypical peers. Children with ASD have higher sugar intake between meals, thus contributing to this problem, and they additionally exhibit poorer oral hygiene habits, greater gingival inflammation, and a greater risk of TDIs than neurotypical children. These results are consistent with findings from previous research from several countries reporting a high prevalence of dental caries and periodontal disease.^4^ However, another systematic review has suggested that ASD is not a predisposing factor for oral diseases and other pathologies.^28^ One previous study has examined oral care among children with ADS in KSA.^29^ The findings in this review might reflect our study design focused solely on the Saudi population. Cultural and socio-demographic differences among the studied populations might have contributed to the contrasting results.

Many children with ASD have sensory sensitivities that make oral hygiene practices uncomfortable or distressing. Consequently, these children may struggle to maintain proper oral hygiene routines, including brushing and flossing. Accessing dental care can be difficult for children with ASD, because of difficulties in tolerating dental visits. These factors underscore the importance of close collaboration among parents, caregivers, and dental professionals to promote good oral health habits and address dental issues promptly. Therefore, a nuanced approach is required to manage dental patients with ASD.^30^ Dentists should be trained adequately accordingly. However, the results of our meta-analysis indicated substantial parental concern regarding the oral health of children with ASD. This finding presents an opportunity to educate parents through specialized programs and other initiatives.

Improving the oral health of children with ASD in KSA will require a tailored approach considering their specific needs and challenges within the cultural context. Sensory sensitivities can be addressed by gradually introducing oral care tools and products with flavors and textures that are acceptable to the child. Visual aids, social stories, and desensitization techniques can help children understand and become more comfortable with oral hygiene routines. Establishing a consistent oral hygiene routine, using positive reinforcement, and incorporating specialized oral care tools can also be beneficial. In addition, promoting a balanced diet low in sugar and attendance at regular dental check-ups is essential. Collaborating with pediatricians, dentists, and other healthcare professionals who understand the cultural context and the specific needs of children with ASD will be crucial for improving their oral health and overall well-being in KSA.

ASD is characterized by a wide range of symptoms and severity levels, and this heterogeneity within the ASD population has not been adequately represented in such studies. Consequently, the findings might not accurately reflect the diverse experiences and needs of individuals with ASD. This limited focus hinders the generalizability of outcomes, because recommendations or interventions derived from these studies might not be suitable for individuals across the entire spectrum of ASD severity. Consequently, incomplete understanding of how oral health is affected in Saudi children with ASD has led to knowledge gaps and hindered the development of effective interventions.

The results of this review should be interpreted with caution for several reasons. First, only three studies compared children with versus without ASD.^10^^,^^16^^,^^21^ In addition, the outcomes were not adjusted in most studies.^10^, ^11^, ^12^^,^^19^^,^^21^, ^22^, ^23^ Both these aspects might have significantly influenced the results of our systematic review, as well as the generalizability and reliability of the findings. The small number of studies might not have provided sufficient evidence to draw robust conclusions regarding the association between ASD and oral health outcomes. The lack of outcome adjustment in most studies might have introduced bias and confounding variables potentially affecting the validity of the results. In the absence of adjustment for potential confounders, such as age, sex, socioeconomic status, or oral health behaviors, the observed associations between ASD and oral health outcomes might have been influenced by factors other than ASD itself.

Furthermore, in this meta-analysis, significant heterogeneity was observed across the included studies; therefore the results should be interpreted with caution. This high heterogeneity was likely to have been influenced by differences in study settings, population characteristics, and measurement tools across the included studies. These factors, along with the limited number of included studies, underscore the need for caution in interpreting the results and highlight the importance of considering these variables in future research. Additionally, our findings demonstrate the need for more research with standardized methods to better understand parental perceptions or caregivers' concerns regarding oral-health-related quality of life in children with ASD.

Consequently, the conclusions drawn from the systematic review are limited in reliability and generalizability. These limitations must be acknowledged, and the findings of the systematic review must be interpreted with caution. Further research with larger sample sizes and rigorous method is necessary to better understand the relationship between ASD and oral health outcomes.

## Conclusions

Despite the limitations of this review, we found that Saudi children with ASD generally have poorer oral health status than their neurotypical peers. However, because of the insufficient number of comparative studies, the oral health status among children with versus without ASD remains unknown. Current and future dentists should be trained accordingly to manage patients with ASD effectively.

## Source of funding

This research did not receive any specific grant from funding agencies in the public, commercial, or not-for-profit sectors.

## Conflict of interest

The authors have no conflict of interest to declare.

## Ethical approval

The authors confirm that this review was conducted in compliance with the guidelines and standards set by the Committee on Publication Ethics (COPE). Because of the nature of this review, IRB approval was not required.

## Authors contributions

Conceptualization, FYA and EK; methodology, FYA and EK; data extraction, EK and FYA; overall validation, MT; formal analysis, FYA and EK; writing—original draft preparation, FYA; writing—review and editing, FYA, MT and EK; supervision, MT and EK; project administration, MT and EK. All authors have critically reviewed and approved the final draft and are responsible for the content and similarity index of the manuscript.

## Data Availability

The data that support the findings of this study are available from the corresponding author upon reasonable request.
