# Supplementary material for: Oral health status of children with autism spectrum disorder in KSA: A systematic review and meta-analysis
Source: J Taibah Univ Med Sci. 2024 Sep 20;19(5):938–46. doi: 10.1016/j.jtumed.2024.09.005 (PMC11470289; doi:10.1016/j.jtumed.2024.09.005)
Supplement: Multimedia component 1 [file mmc1.docx]

Appendix 1

**Search terms used in the included Databases.**

PubMed

| **Search** | **Query** | **Results** |
| --- | --- | --- |
| #2 | Search: **(("oral"[All Fields] OR "DMFT"[All Fields] OR "DMFT"[All Fields] OR "dental caries"[All Fields] OR "periodontal disease"[All Fields] OR "periodontitis"[All Fields] OR "gingivitis"[All Fields] OR "gingival disease"[All Fields] OR "plaque index"[All Fields] OR "gingival index"[All Fields] OR "periodontal disease index"[All Fields] OR "oral health related quality of life"[All Fields] OR "pocket depth"[All Fields] OR "oral disease"[All Fields] OR "oral inflammation"[All Fields] OR "malocclusion"[All Fields] OR "traumatic dental injuries"[All Fields]) AND ("autism"[All Fields] OR "autism spectrum disorder"[All Fields] OR "autistic"[All Fields] OR "disabilities"[All Fields] OR "disability"[All Fields] OR "special needs"[All Fields] OR "special care"[All Fields] OR "special health care needs"[All Fields]) AND ("Saudi Arabia"[All Fields] OR "Kingdom of Saudi Arabia"[All Fields]))** | 226 |

Embase

| **Search** | **Query** | **Results** |
| --- | --- | --- |
| #1 | **("oral" OR "DMFT" OR "DMFT" OR "dental caries" OR "periodontal disease" OR "periodontitis" OR "gingivitis" OR "gingival disease" OR "plaque index" OR "gingival index" OR "periodontal disease index" OR "oral health related quality of life" OR "pocket depth" OR "oral disease" OR "oral inflammation" OR "malocclusion" OR "traumatic dental injuries")**  **AND**  **("autism" OR "autism spectrum disorder" OR "autistic" OR "disabilities" OR "disability" OR "special needs" OR "special care" OR "special health care needs")**  **AND**  **("Saudi Arabia" OR "Kingdom of Saudi Arabia")** | 403 |

Web of Science

| **Search** | **Query** | **Results** |
| --- | --- | --- |
| #2 | **TS=(("oral" OR "DMFT" OR "DMFT" OR "dental caries" OR "periodontal disease" OR "periodontitis" OR "gingivitis" OR "gingival disease" OR "plaque index" OR "gingival index" OR "periodontal disease index" OR "oral health related quality of life" OR "pocket depth" OR "oral disease" OR "oral inflammation" OR "malocclusion" OR "traumatic dental injuries")**  **AND**  **("autism" OR "autism spectrum disorder" OR "autistic" OR "disabilities" OR "disability" OR "special needs" OR "special care" OR "special health care needs")**  **AND**  **("Saudi Arabia" OR "Kingdom of Saudi Arabia"))** | 62 |

Scopus

| **Search** | **Query** | **Results** |
| --- | --- | --- |
| #2 | TITLE-ABS-KEY **(("oral" OR "DMFT" OR "DMFT" OR "dental caries" OR "periodontal disease" OR "periodontitis" OR "gingivitis" OR "gingival disease" OR "plaque index" OR "gingival index" OR "periodontal disease index" OR "oral health related quality of life" OR "pocket depth" OR "oral disease" OR "oral inflammation" OR "malocclusion" OR "traumatic dental injuries")**  **AND**  **("autism" OR "autism spectrum disorder" OR "autistic" OR "disabilities" OR "disability" OR "special needs" OR "special care" OR "special health care needs")**  **AND**  **("Saudi Arabia" OR "Kingdom of Saudi Arabia"))** | 72 |

Additional Searches

Google Scholar (up to the first 20 pages.)

| **Search** | **Query** |
| --- | --- |
| #2 | **Search String:**  **("oral" OR "DMFT" OR "dental caries" OR "oral health related quality of life" OR "gingival disease" OR "malocclusion" OR "traumatic dental injuries") AND ("autism" OR "disabilities") AND "Saudi Arabia"** |
